# Current engagement with unreliable sites from web search driven by navigational search

**DOI:** 10.1126/sciadv.adn3750

**Published:** 2024-10-30

**Authors:** Kevin T. Greene, Nilima Pisharody, Lucas Augusto Meyer, Mayana Pereira, Rahul Dodhia, Juan Lavista Ferres, Jacob N. Shapiro

**Affiliations:** ^1^ Empirical Studies of Conflict Project, Princeton University, Princeton, NJ, USA.; ^2^ AI for Good Research Lab, Microsoft, Redmond, WA, USA.; ^3^ University of Brasilia, Brasilia, Brazil.

## Abstract

Do search engine algorithms systematically expose users to content from unreliable sites? There is widespread concern that they do, but little systematic evidence that search engine algorithms, rather than user-expressed preferences, are driving current exposure to and engagement with unreliable information sources. Using two datasets totaling roughly 14 billion search engine result pages (SERPs) from Bing, the second most popular search engine in the U.S., we show that search exposes users to few unreliable information sources. The vast majority of engagement with unreliable information sources from search occurs when users are explicitly searching for information from those sites, despite those searches being an extremely small share of the overall search volume. Our findings highlight the importance of accounting for user preference when examining engagement with unreliable sources from web search.

## Teaser

Search engines seldom lead unaware users to unreliable sources; most engagement occurs when users seek out these sources.

## INTRODUCTION

Search engines play a key role in how people access information, being used by most Americans (*1*) as a primary referrer to information sources (*2*–*4*). There are growing concerns in popular media outlets that search engines’ algorithms are undermining democracy by directing users to unreliable information (*5*–*8*). In recent years, online misinformation has arguably undermined trust in U.S. elections (*9*), hampered public health efforts to combat the COVID-19 pandemic (*10*, *11*), and fueled a violent insurrection at the U.S. Capitol (*12*, *13*). Two key concerns are that online platforms expose unwitting users to unreliable information through algorithmic recommendation systems (*14*–*16*) and that these platforms allow users to easily navigate to and engage with information from unreliable sites (*2*–*4*, *17*).

These problems are particularly acute for search engines as they are used daily by most Americans (*18*), are a highly trusted gateway to information (*19*, *20*), and have the potential to impact electoral outcomes (*21*, *22*). Past investigations have found some support for these concerns. Several studies have conducted algorithm audits where a sample of keywords was fed into a search engine and the returned results were evaluated. This work has found that search engines expose users to a considerable amount of politically biased (*23*–*27*) or unreliable sites (*28*–*31*). Moving beyond studies of theoretical exposure, Robertson *et al.* (*32*) survey the search behavior of a large sample of real users during the 2018 and 2020 US elections. They find that despite being exposed to a variety of sources users favored content that aligned with their ideological views. The study highlights the role that user choices play in interactions with partisan information sources and echoes similar findings on other online platforms (*14*–*16*, *33*, *34*). In the Supplementary Materials, we provide additional information on previous studies.

However, there are substantial gaps in our understanding of the role search engines play in the exposure to and engagement with unreliable sources of information. First, other than (*32*), previous work has generally studied engagement and exposure to unreliable sources separately. Studies of exposure to unreliable sources capture the search results returned from small samples of keywords (typically fewer than 20) but are unable to measure how often these keywords lead to engagement with unreliable sites. Studies of engagement (*2*–*4*) capture user movement from search engines to unreliable domains, but do not have information on the full set of links supplied to users by search engines. Second, past work has not collected representative samples of the information returned by search engines (*6*), instead often selecting on the dependent variable by investigating only search queries related to conspiracy theories or misinformation narratives (*8*). Last, despite growing evidence that engagement with unreliable sources on other social media platforms is driven by user choices rather than algorithms (*14*–*16*, *33*, *34*), aside from (*32*), previous work has not explicitly compared the roles of user choice and search algorithms in leading users to unreliable sites.

To fill these gaps, we collect two large random samples of anonymized search requests from the Bing search engine to conduct what is, to our knowledge, the first large-scale, representative audit of the content provided by search.

Sample 1, designed to reflect what a typical user is likely to view, contains all the results returned between June and August 2022 for a weighted random sample of more than 100,000 information-seeking queries. We define such queries as the set of all queries that at least once exposed users to any of the more than 8000 domains rated for reliability by NewsGuard (roughly 5000 reliable and 3000 unreliable sites), whose ratings cover the majority of news information consumed online (*35*) and are widely used in academic research (*36*). This provides a population of queries that could lead users to either reliable or unreliable information sources and does not require us to use a set of keywords that would likely be unrepresentative of real-world user behavior. A query’s likelihood of selection was proportional to the number of times it was searched. This approach prioritizes heavily searched queries with the potential for greater impact and contains roughly 12.6 billion search engine result pages (SERPs). This sample was collected before the integration of large language models into the search engine.

Sample 2, designed to broadly characterize the search information environment, is a simple random sample of search results collected between April and June 2023, leading to the same set of sites as sample 1. This sample, collected after the integration of large language models into the search engine, contains roughly 1.1 billion SERPs. As search engine results are long-tailed distributions, sample 2 provides a representative sample of the population of search results but largely features queries that were seldom searched, while sample 1 provides a representative sampling of heavily searched queries. In the Supplementary Materials, we provide additional information about these samples and our sampling strategies.

To measure exposure, we collect the URLs of the first 10 results returned through Bing for each search (roughly 14 billion SERPs). The top 10 results were selected as few users move past the first page of results (*37*). To measure engagement, we capture nearly 900 million instances where a user navigated to a link presented by the search engine.

To evaluate the role of user preferences in the exposure to and engagement with domains rated by third parties as unreliable information sources, we distinguish between “navigational queries” (*38*), where users are clearly seeking out specific sites and more general informational queries. Navigational searches are a well-established concept in information science (*38*, *39*). An example would be a user seeking the website for National Public Radio searching for “NPR.” For our study, we focus specifically on unreliable site navigational queries (USNQs). Searches for the names of unreliable sites are clear instances of users seeking out information sources rated by NewsGuard as unreliable. In the Supplementary Materials, we provide additional examples of USNQs and detail our approach to identifying them accurately at scale.

Results from both samples of search results indicate that for general informational searches, search exposes users to few unreliable information sources. Further, we find consistent evidence that user preferences play an important role in both exposure to and engagement with unreliable sites from search. By identifying USNQ searches, searches where users specifically search for the name of an unreliable domain, we are able to directly measure the role of user preferences in engagement with unreliable domains. While USNQ make up a small portion of the total searches, they account for more than 82% of the total engagement with unreliable sites. Overall, our results indicate that individual choices are driving engagement with unreliable information sources from search, rather than algorithms.

These findings inform long-standing questions about the current role of search engines in exposing and leading users to unreliable information sources. In particular, they show that meaningful evaluations of search engines’ impact on engagement and exposure to unreliable sources require researchers to explicitly evaluate the role of user choice consistent with previous work on other platforms (*14*–*16*, *32*–*34*). However, our findings are limited to current engagement with unreliable sites in web search and do not address the broader role of search algorithms and the online information environment in shaping today’s user preferences.

## RESULTS

**Fig. 1.**
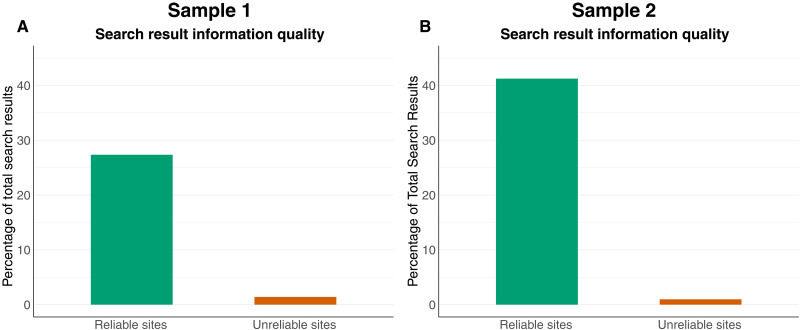
Exposure to unreliable and reliable sources. Each bar represents the percentage of the total search results that link to unreliable or reliable sites. (**A**) presents results from a weighted random sample of search results that capture highly searched queries that a typical user is likely to view, while (**B**) presents results from a random sample of search results that broadly characterize the search information environment.

We find that exposure to unreliable sites from Bing is infrequent (Fig. 1). In sample 1, around 1.4% of SERPs linked to an unreliable news source and 27% to reliable news sites. In sample 2, around 0.9% of SERPs linked to an unreliable news source and 41% to a reliable news site. This means that Bing exposed users to between 19 (sample 1) and 45 (sample 2) times more reliable information sources than unreliable information sources for searches on terms that led at least once to sites in our sample.

We also assess whether unreliable sites are systematically returned at higher ranks. As higher ranked search results receive more attention from users (*37*), having unreliable sites at higher result ranks may lead to more navigation to those sites.

**Fig. 2.**
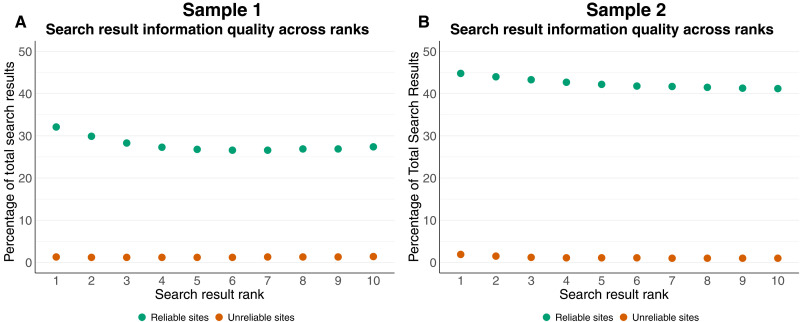
Exposure to unreliable and reliable information sources across search result ranks. Each dot represents the percentage of the total search results that link to unreliable or reliable sites. The color of the points indicates the information source. Each value includes its corresponding 95% confidence interval. However, they are too small to be visible in the plot. (**A**) presents results from a weighted random sample of search results that capture highly searched queries that a typical user is likely to view, while (**B**) presents results from a random sample of search results that broadly characterize the search information environment.

The percentage of SERPs linking to unreliable and unreliable information sites returned up to rank *k* is shown in the left of Fig. 2. In sample 1, around 1.2% of the top 3 results link to unreliable information sites, compared to 1.4% overall. For reliable sites, there is also little substantive difference, as we find 28% of the top 3 results link to reliable sources compared to 27% overall. In sample 2, around 1.2% of the top 3 results link to unreliable information sites, compared to 0.9% overall. For reliable sites, there is also little substantive difference, as we find 43% of the top 3 results link to reliable sources compared to 41% overall. Overall, unreliable news sources rarely appear in the top results.

We next study whether exposure to unreliable information sources is due to Bing’s search algorithm delivering unreliable information sources to unsuspecting users or to users seeking out domains rated by third parties as unreliable information sources. To account for the role of user preferences, we define USNQs as queries that indicate users are specifically seeking out a domain rated as an unreliable source (i.e., searching for “breitbart” when seeking breitbart.com). We identify these as queries that frequently led to the landing page of an unreliable site. An evaluation of our approach using domain experts indicates that we can effectively identify USNQs (precision = 0.986 and recall = 0.915). Our approach is discussed in greater detail in the Supplementary Materials.

We begin by providing information on the occurrence of USNQ searches (Table 1). Overall, USNQ searches make up a small percent of the total searches, 0.88% in sample 1 and 1.62% in sample 2. However, despite being relatively rare, 46.98% of the total exposure to unreliable sites originate from USNQ searches in sample 1 and 15.37% in sample 2. This indicates that a disproportionate amount of exposure to unreliable sites arise from instances where users are seeking out these sources. User preferences are even more strongly associated with engagement with unreliable sites. For example, in sample 2, USNQ searches account for less than 2% of searches, yet more than 82% of the engagement with unreliable sites originate from a USNQ search (Table 1). Put another way, users seldom unexpectedly engage with unreliable sites from search, instead, almost all engagement from search occurs when users are specifically seeking it out by searching for the name of an unreliable site.

**Table 1.** Descriptive information on the occurrence of USNQ searches. % Total searches indicates the percentage of total search results that are USNQ searches. % Unreliable exposure indicates the percentage of total exposure to unreliable sites originating from USNQ searches. % Unreliable engagement indicates the percentage of total engagement with unreliable sites originating from USNQ searches.

	% Total searches	% Unreliable exposure	% Unreliable engagement
Sample 1	0.88	15.37	82.21
Sample 2	1.62	46.98	82.03

**Fig. 3.**
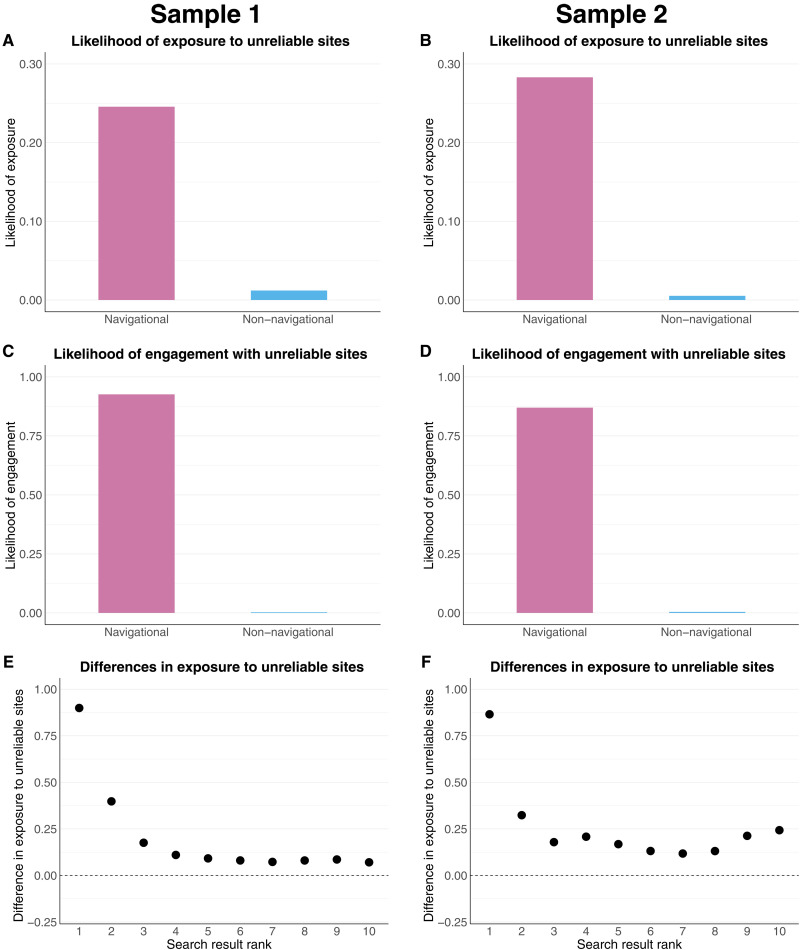
Likelihood of exposure to and engagement with unreliable information sources for USNQ and non-USNQ searches. Engagement is the probability of engaging with unreliable sources conditional on the search query type. Exposure is the probability of being exposed to unreliable sources conditional on the search query type. (**A** and **B**) The likelihood of exposure to unreliable sources across search types. (**C** and **D**) The likelihood of engagement with unreliable sources across search types. (**E** and **F**) Differences in exposure to unreliable sources at each result rank across search types. Sample 1 presents results from a weighted random sample of search results that capture highly searched queries that a typical user is likely to view, while sample 2 presents results from a random sample of search results that broadly characterize the search information environment.

Next, we provide information on the probability of being exposed to or engaging with unreliable sites for USNQ and non-USNQ searches (Fig. 3). First, the likelihood of being exposed to unreliable sites is considerably higher for USNQ searches than non-USNQ searches (Fig. 3, A and B). In sample 1, exposure is 20 times as likely for USNQ searches, while exposure is 54 times as likely in sample 2. In Fig. 3 (E and F), we calculate the difference in the proportion of unreliable sites featured at ranks 1 through 10 for USNQ and non-USNQ searches. Across both samples, we find that particularly at ranks 1 and 2, there is considerably more exposure to unreliable sites from USNQ searches. For non-USNQ searches, less than 1% of the results at rank 1 were unreliable sites, while this value was around 90% for USNQ searches.

The probability of engaging with unreliable sites is also considerably higher for USNQ relative to non-USNQ queries. In sample 1, the likelihood is 0.926 for USNQ queries, compared to 0.003 for non-USNQ queries. In sample 2, the likelihood is 0.870 for USNQ queries, compared to 0.004 for non-USNQ queries. Overall, arriving at unreliable sites from search is a rare occurrence for anyone not specifically seeking out this type of information.

To ensure that our conclusions are not driven purely by NewsGuard’s definitions of unreliable sites, we re-estimate our results using another organization that evaluates the quality of sites, Media Bias/Fact Check (MBFC). The conclusions from these analyses are consistent with those previously presented and can be found in the Supplementary Materials.

## DISCUSSION

This work informs long-standing questions about the current role of search engines in exposing and leading users to unreliable information sources. Despite previous suggestions, we find that for general informational searches, search exposes users to few unreliable information sources. Further, user preferences appear to play an outsized role in both exposure to and engagement with unreliable sites from search. While USNQ searches are a small portion of the total searches, they account for more than 82% of the total engagement with unreliable sites. Overall, it appears that individual choices are driving engagement with unreliable information sources from search, not algorithms. We find consistent results when evaluating a random sample of search results that broadly characterize the information environment and a weighted random sample of search results that capture highly searched queries that a typical user is likely to view, further suggesting that our results are not driven by either rare queries or sets of popular searches that may not be broadly representative. While our study uses data that capture the real-world activity of users, we find results consistent with (*32*).

Our work has several implications for research on the information returned by search engines. First, making valid inferences about the overall exposure to unreliable sites requires researchers to include search terms that do not lead to unreliable sites. Past work has frequently selected on the dependent variable by measuring the amount of unreliable sites presented exclusively from search queries related to conspiracy theories or misinformation narratives. Second, researchers should explicitly compare the roles of user choice and search algorithms in leading users to unreliable sites, consistent with recent work on social media platforms (*14*–*16*, *32*). If, as we find, engagement with unreliable sites is largely associated with user choice, focusing solely on the search algorithms is unlikely to produce successful policy solutions. Last, while not the focus of this research, our results suggest that the role of USNQ in engagement with unreliable sources has not changed after the introduction of large language models into Bing. As LLMs are being increasingly used, a more comprehensive study of their impact on search quality and user behavior is warranted.

Our work also has several implications for improving the quality of information returned by search engines. First, while others have noted the impact of query type on the quality of information returned (*32*, *40*), our findings suggest that increased attention should be paid to the role of USNQ in unreliable information exposure and engagement. This could be done by including USNQ in existing news quality signals. Our findings further suggest that incidental exposure to unreliable sites from search is rare, and engagement extremely so. Reducing the exposure and engagement with unreliable sites likely requires interventions targeted toward individuals seeking out unreliable sources (*36*, *41*). On the basis of our findings, further downranking unreliable sites in search is unlikely to markedly affect engagement with unreliable information. One solution might be upranking reliable sites and fact-checks for searches seeking out unreliable sources. This will expose users to a different set of information that may expose them to a wider set of information (*42*, *43*).

Some limitations of our study should be noted. First, our analyses were conducted using data from the Bing search engine; thus, we cannot say that our results hold for search engines overall. However, as past work has suggested that Bing exposes users to more unreliable information sources than other search engines (*6*, *29*, *30*), it is unlikely that an analysis using another search engine would conclude that exposure to unreliable sites is driven primarily by algorithms rather than users. Further, we have no reason to believe that the number of USNQ searches would be larger when using a different search engine. However, our analyses should be replicated using data from other search engines. Second, while we find that arriving at an unreliable domain is overwhelmingly the result of a search query including the name of a specific unreliable site, this does not mean that search engines play no role in users arriving at unreliable domains. As noted by others (*3*), search engines are a major referrer to unreliable domains. Further assessments of potential differences between algorithmic recommendation systems could contribute to future research. Third, as our study does not track the actions of individuals, we are unable to evaluate other explanations, for instance, if users were recommended unreliable domains in the past. In particular, the role of algorithms in shaping user preference is an important remaining question for future research. Fourth, while we collected representative samples of searches, some specific topical domains might present more (or less) exposure to unreliable sites. This should be further assessed in future work. Last, consistent with previous work (*44*), our analyses rely on measures of reliability at the domain level. We cannot identify if a specific URL contains unreliable information, only that it comes from a domain that has been rated as an unreliable information source by a third party.

## MATERIALS AND METHODS

### Unreliable domains

Domains are classified using two different groups that rate the quality of online news sources. The first is NewsGuard (*35*). NewsGuard is a company that rates the journalistic quality of news websites and has been widely used in academic research (*36*). Their ratings are based on a set of nine criteria, including if sites publish false content, correct factual errors, and effectively separate news from opinion. Sites that receive an overall score that falls below 60 of 100 are classified as unreliable information sources. Domains with a score of 60 or greater are classified as reliable information sources.

The second is MBFC. MBFC is a site rating the quality of online news sources. MBFC evaluates the quality of factual reporting of sites on a 10-point scale. Sites with a score of 7 or higher are rated as “low” or “very low” in regard to their reporting. MBFC notes that low-rated domains rarely use credible sources and are not reliable sources of information. While past work has shown high agreement between distinct lists of unreliable news sources (*45*), both are included to ensure that our results are not driven by any single organization’s definition of unreliable sites.

### Search result data

From the population of Bing searches, we narrow our potential sample down to information-seeking queries, which we define as the set of queries that led users to any of the more than 8000 domains rated for reliability by NewsGuard (*35*). These provide a massive dataset but help filter out searches that solely relate to entertainment or online shopping. Using this procedure, we select two random samples of search results. These large random samples of SERPs help mitigate the potential influence of geographic differences in search results or search personalization (*23*, *46*) and allow us to draw conclusions about the quality of information provided by search.

Our first sample was collected between April and June 2023 and contains roughly 1.1 billion SERPs. The sample was collected by randomly sampling search results from all information-seeking English queries, after excluding bots and adult queries. This sample provides representative details about the quality of information available from informational searches from Bing. Our second sample was collected between June and August 2022 and contains roughly 12.6 billion SERPs. The sample was collected by taking a weighted random sample of more than 100,000 information-seeking English queries, after removing bots and adult queries and collecting all the search results they returned during this period. The likelihood of selection was proportional to a query’s total search volume. This sample was collected because the distribution of search engine results is long-tailed, with a large amount of the search volume being concentrated in a relatively small number of queries (*47*). By randomly sampling queries weighted on the basis of their overall search volume, we can provide better coverage of highly searched queries reflecting the information that a typical user of Bing might be exposed to. In both samples, we collect the top 10 search results as few users are likely to engage with content at higher ranks (*37*). All results were anonymized, removing any personally identifying information.

### Measuring exposure and engagement

We define exposure as the search results retrieved that are presented on a user’s screen. Specifically, for our analyses, these are the top 10 URLs supplied by Bing for each search. This definition aligns closely with those used by (*32*, *33*). We define engagement as navigation/visits to a page that was presented by the search engine. For example, if a user were presented with the URL for https://esoc.princeton.edu and followed that URL to the webpage, this would count as an engagement. While there are many ways that a user might engage with a search engine, for our study, the salient actions are movements to URLs that were presented. Our definition of engagement most closely aligns with the concept of “following” from (*32*), moving from one page to another via hyperlink.

For each search result, we collect the top 10 SERPs presented. We also recorded if the user engaged with any of the presented links. In addition, we record the search result rank for each SERP. For each SERP, we know the URL presented, its result rank (1 to 10), and if the link was engaged with. As our analyses focus on the quality of information presented, these data are combined with the domain quality ratings provided by NewsGuard and MBFC. Specifically, we truncate each URL to the second-level domain name. The URL https://reuters.com/markets/us/futures-rise-us-congress-averts-govt-shutdown-2023-10-02/ would resolve to reuters.com. Each domain is then checked against the lists compiled by the rating agencies, providing domain-level measures of the quality of information, consistent with previous work (*32*, *48*).

### Unreliable site navigational queries

To identify USNQs, we collected all search queries for May 2022 that users used to navigate to web pages. We consider queries to be USNQ if they tend to direct users to the home page of an unreliable news site. Specifically, we identify queries that direct to the root page of an unreliable domain more than 80% of the time, and that are searched by more than 1000 users. For example, “breitbart” and “bright bart” were frequently used by users as shortcuts to navigate to breitbart.com. In our analysis, we label queries as USNQ if they contain these previously identified navigational phrases. For example, the query “fbi corruption report bright bart” would be labeled as USNQ, whereas the query “is bart simpson bright” would not, because it does not contain a valid navigational phrase.

## Supplementary Materials

### This PDF file includes:

Materials and Methods Figs. S1 to S5 Tables S1 to S7 Supplementary Text References
