# Supplementary material for: Current engagement with unreliable sites from web search driven by navigational search
Source: Sci Adv. 2024 Oct 30;10(44):eadn3750. doi: 10.1126/sciadv.adn3750 (PMC11524180; doi:10.1126/sciadv.adn3750)
Supplement: Supplementary file 1 — Materials and Methods Figs. S1 to S5 Tables S1 to S7 Supplementary Text References [file sciadv.adn3750_sm.pdf]

Science Advances
AAAS

# Supplementary Materials for

## Current engagement with unreliable sites from web search driven by navigational search

Kevin T. Greene *et al.*

Corresponding author: Kevin T. Greene, kg2082@princeton.edu

*Sci. Adv.* **10**, eadn3750 (2024)
DOI: 10.1126/sciadv.adn3750

**This PDF file includes:**

Materials and Methods
Figs. S1 to S5
Tables S1 to S7
Supplementary Text
References

# Materials and Methods

## Search Result Data

Descriptive details on our samples are presented in Table S1.

| Sample | Sample Type | SERPs | Queries | URLs | Domains |
|---|---|---|---|---|---|
| Sample 1 | random | 1,107,910,744 | 10,855,084 | 38,694,081 | 2,208,849 |
| Sample 2 | weighted random | 12,576,216,317 | 118,025 | 11,438,039 | 1,350,178 |

Table S1: Descriptive information for our samples of search result.

We utilize two different sampling approaches to evaluate the exposure to and engagement with unreliable domains from search. As search results are long-tailed distributions, Sample 1 is representative but largely features queries that were seldom searched, while Sample 2 provides a representative sampling of heavily searched queries. These compositional differences also somewhat drive the differences in the overall percentage of exposure to unreliable sites from navigational searches (Main paper, Table 1). Queries in Sample 1 will be considerably rarer than queries present in Sample 2. Second, there are considerably more navigational searches in Sample 1 relative to Sample 2, meaning they account for a higher percentage of the total queries. Finally, in Sample 1 for navigational searches, unreliable sites show up more often at higher ranked results (9 and 10).

## Additional Details on Sampling Approaches

As we noted previously we utilize two different sampling approaches to evaluate the exposure to and engagement with unreliable domains from search. As search results are long-tailed distributions, the simple random sample is representative but largely features queries that were seldom searched, while the weighted provides a representative sampling of heavily searched queries. In this section, we conduct a simulation to better illustrate how the two sampling approaches differ from one another. The simulation has the advantage of not being influenced by potential over time differences in samples that might make them incomparable.

The distribution of search query frequency is simulated using the Zipf distribution. Past work has found that search results exhibit a power-law distribution (*47*), which can be represented by the Zipf. We used the implementation in Python through numpy. We set the alpha parameter to 1.68 and created 100,000 samples. The distribution is presented in Figure S1. As expected, most of the density of the distribution is concentrated at low numbers of searches. The median value is 2.

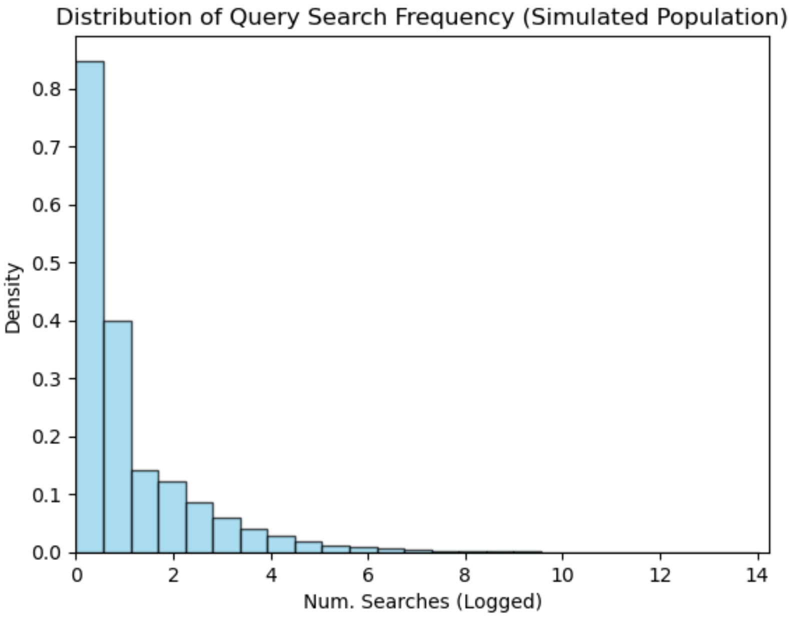


Figure S1: Distribution of total (logged) searches for a simulated population of queries. The population was generated using 100,000 samples from the Zipf distribution with alpha 1.68.

From this distribution, we create a simple random sample by randomly sampling 2.5% of the total results. To create a weighted random sample, for each query, we calculate a set of weights, where the weights are proportional to how often the query was searched in the population. We then randomly sample all the search results for 500 queries. The probability of selection is equal to the query's weight. The distributions for each sampling approach are presented in Figure S2. The figure illustrates that each approach focuses on different parts of the population. The simple random sample is representative of the population and thus most of the results are drawn from queries that were seldom searched. On the other hand, the weighted sample is much more likely to select queries that were heavily searched. The trade-off is thus broad representativeness for potential impactfulness. Table S2 provides descriptive results to further illustrate this point. In particular, the last column indicates the percent of queries that were searched 5 or fewer times. For the simple random sample, 86% of the queries were searched 5 or fewer times, while this value is 3% for the weighted random sample.

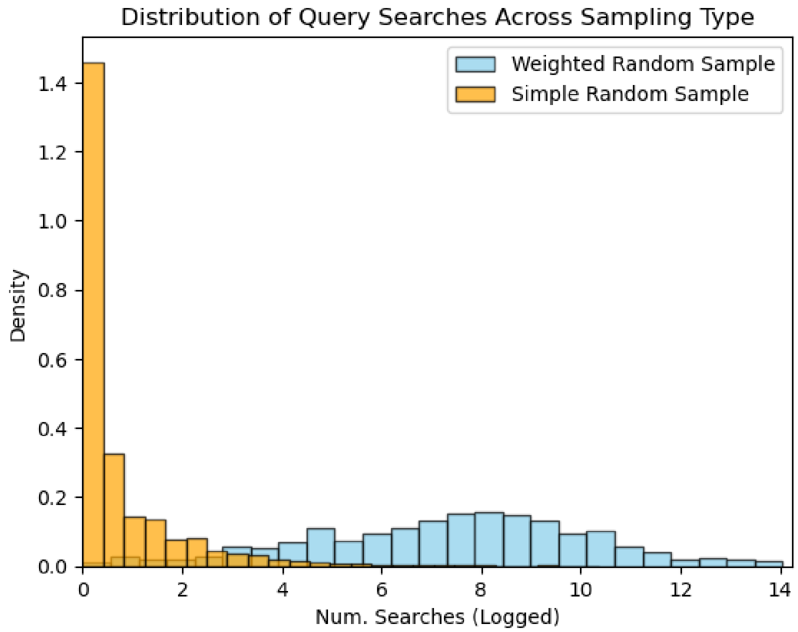


Figure S2: Distributions of total (logged) searches for the simple random and weighted random samples.

| Sample Type | Min | Max | Mean | Median | % <= 5 |
|---|---|---|---|---|---|
| simple random | 1.00 | 31,469.00 | 32.69 | 1.00 | 86.0 |
| weighted random | 1.00 | 1,261,459.00 | 29,652.45 | 1,840.00 | 3.0 |

Table S2: Descriptive information for a simple and weighted random sample from a simulated population of queries. The last column (% <= 5) indicates the percent of queries in the sample that were searched five or fewer times.

## Navigational Queries

In this section, we provide additional information about navigational searches, our approach to accurately identifying them at scale, and how they can influence the quality of information returned by search engines.

### Identifying Navigational Queries

In earlier browsers, users would type the URL for the website that they wanted to navigate to in an element called the "address bar." Modern browsers like Google Chrome, Microsoft Edge, and Apple Safari have combined the address bar with the ability to run searches. The resulting element is called the "omnibox." When a user types an existing URL in the omnibox, the browser simply navigates to that URL. When the user types something that is not formatted as a URL the browser performs a search using the default search engine.

Users frequently employ the omnibox as a shortcut to navigate to their most used sites. For example, instead of typing the URL https://www.nytimes.com, a user might simply enter "nyt" in the omnibox. This will execute a search for "nyt" in the default search engine, and in both Google and Bing, the first result (and likely several of the top results) will be the website for the *New York Times*. Users employ a similar mechanism to direct the search engine to provide a specific webpage. For example, a user wanting to see political news from Reuters might enter "political news reuters" in the omnibox. Queries that contain clear directions to specific sites are frequently called "navigational queries" (*38, 39*). For our study navigational queries provide powerful leverage in untangling the role of search algorithms and user preferences. Users entering navigational searches that contain the name of an unreliable domain, represent clear evidence of users seeking out unreliable information sources. In Table S3 we provide examples of popular navigational and non-navigational queries from our samples. There are potentially two types of navigational queries, "pure navigational queries, that contain only the name of a domain and queries that refer to a domain along with other search terms. We took a random sample of 600 queries and manually evaluated them to determine if they were "pure navigational" (including only the name of a domain or a close misspelling) or queries that included additional content. In total, we find that roughly 53% are "pure navigational" queries.

| Navigational Queries | Non-Navigational Queries |
|---|---|
| brietbart news, breitbart news, breitbart, breitbart website, newsmax, newsmax news, news max, occupy democrats, sputnik news | wimbledon 2022, brittney griner, top news, postage holiday price hike, roe v wade overturned, hoover dam explosion, queen elizabeth ii |

Table S3: Examples of highly searched navigational and non-navigational searches in our samples.

### Validating Navigational Query Measure

While our measure of navigational queries is intuitive, we want to be sure that our measure accurately identifies clear user-driven requests for unreliable sites. To this end, we randomly sample 1000 queries and then have them annotated by a domain expert. Queries that are unambiguous requests for a domain rated as unreliable by NewsGuard are coded as 1 and all other queries are coded as 0. These ground truth measures are then compared against the classifications made by our approach for identifying navigational queries. We evaluate the performance of our approach using precision and recall. Because there are large differences in how often a given query is searched, we also include measures of precision and recall that account for search volume. These results are presented in Table S4. We find that our approach is highly effective at identifying navigational searches. Our approach is especially proficient at avoiding false positives.

| Metric | Score |
|---|---|
| Precision | .987 |
| Recall | .915 |
| Precision (Weighted) | .999 |
| Recall (Weighted) | .927 |

Table S4: Performance of our approach to identifying navigational search queries. Evaluations are based on human annotation of a random sample of 1000 queries.

### Navigational Searches and Information Quality

We provide examples of two searches that reflect popular topics in the entertainment space (Table S5). In our example, searches for both "taylor swift" and "nfl" return no unreliable domains. Most of the results returned are home pages for the person/league or their social media channels. However, after adding a navigational phrase (the name of an unreliable domain) the majority of results link to sites that were rated as unreliable. These examples illustrate that even in the case of popular entertainment focused queries, the addition of the name of an unreliable domain will dramatically change the quality of information returned. These results were collected from the Bing search engine accessed through SerpAPI using the queries presented in Table S5.

While this example does not employ actual user data, and thus we cannot evaluate the impact on engagement, it does help illustrate the dynamics taking place. Users request content from an unreliable site through navigational searches, and the search engine, working correctly, returns the named site at a high result rank. Observing high engagement with unreliable sites is expected because we know that the user was looking for an unreliable site in the first place (they entered a navigational search).

| result rank | taylor swift | taylor swift breitbart | taylor swift newsmax |
|---|---|---|---|
| 1 | taylorswift.com | breitbart.com | newsmax.com |
| 2 | en.wikipedia.org | breitbart.com | newsmax.com |
| 3 | cnn.com | breitbart.com | newsmax.com |
| 4 | biography.com | breitbart.com | newsmax.com |
| 5 | youtube.com | breitbart.com | newsmax.com |
| 6 | instagram.com | breitbart.com | newsmax.com |
| 7 | britannica.com | breitbart.com | newsmax.com |
| 8 | twitter.com | breitbart.com | newsmax.com |

| result rank | nfl | nfl breitbart | nfl newsmax |
|---|---|---|---|
| 1 | foxsports.com | breitbart.com | newsmax.com |
| 2 | nfl.com | breitbart.com | thedailybeast.com |
| 3 | nfl.com | breitbart.com | newsweek.com |
| 4 | betonline.ag | breitbart.com | newsmax.com |
| 5 | youtube.com | breitbart.com | thespun.com |
| 6 | reddit.com | hotair.com | newsmax.com |
| 7 | nfl.com | breitbart.com | newsmax.com |
| 8 | nfl.com | breitbart.com | newsmax.com |

Table S5: Examples of the impact of navigational searches on the quality of search results. Each cell contains the domain returned at a given rank for a given search query. The leftmost queries are related to popular entertainment topics. The queries to the right add the name of a domain rated as unreliable by NewsGuard. Results colored green contain a domain rated as reliable by NewsGuard, and results colored orange contain a domain rated as unreliable by NewsGuard.

## Distribution of Engagement and Exposure Across Unreliable Domains

In Table S3 we present empirical cumulative distribution functions showing the distribution of the engagement and exposure across unreliable domains. Overall, the top 20 domains account for around 85% of the total engagement with unreliable news sites and 84% of the total exposure.

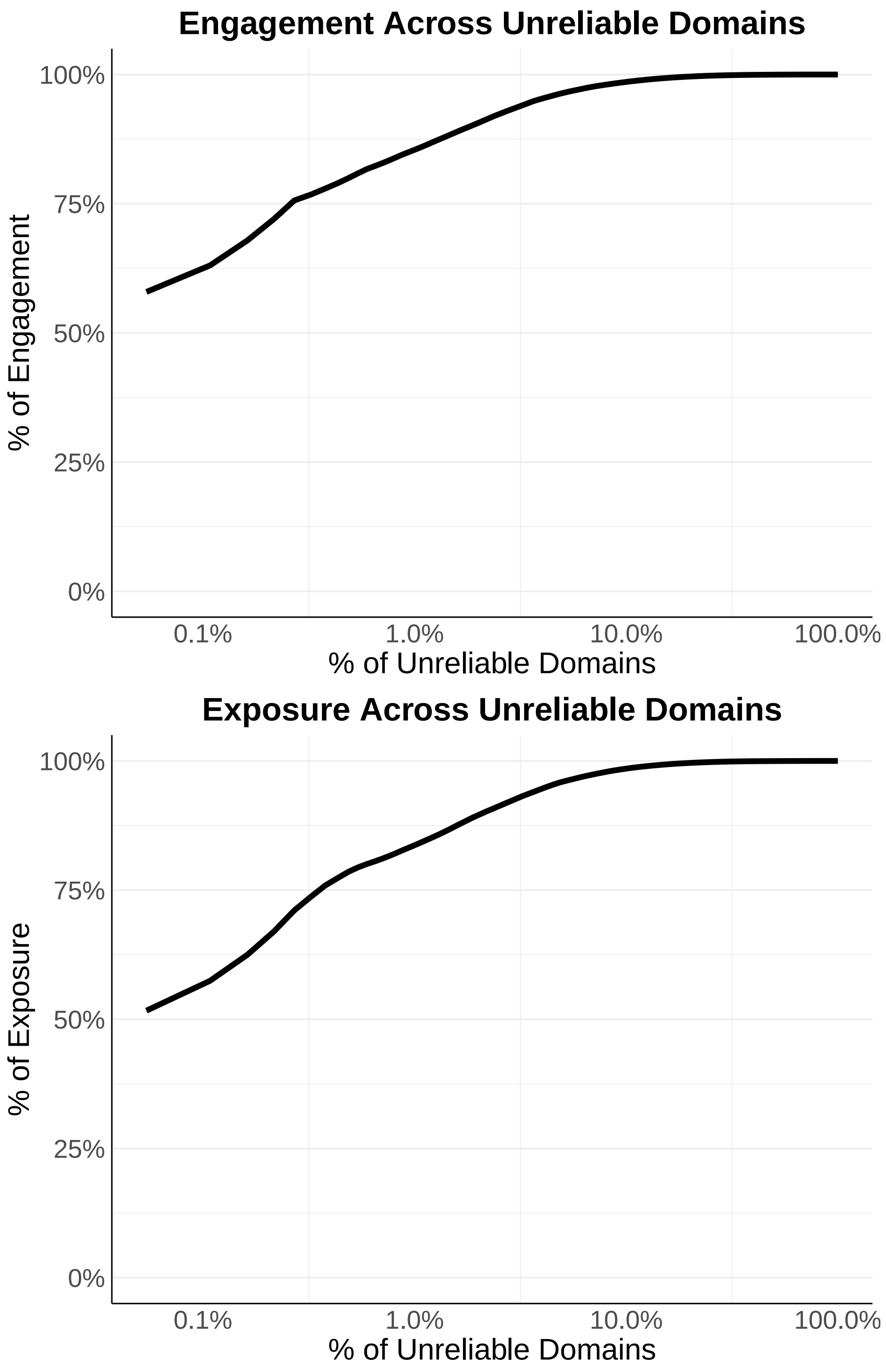


Figure S3: Concentration of the total exposure to and engagement for unreliable domains. Total engagement and exposure are aggregated to the domain level for each of the unreliable domains in the study. Most unreliable domains receive little engagement or exposure.

## Comparison to Other Studies

In Table S6 we provide a summary of the approaches carried about by related studies of exposure to and engagement with unreliable content from search engines. The primary difference between the studies is the methods used. Audits collect information on potential exposure to unreliable content using a predefined list of keywords but do not measure actual exposure to or engagement with this content. Surveys use samples of user browser behavior that allow for measuring user engagement and/or exposure to unreliable content. This study (Real World) provides similar information as the survey approach but features data collected from the "real world", rather than within a survey. This approach allows us to measure 1.) the search results presented to users and 2.) if users navigated from the presented link to the domain. While each approach offers advantages, for our study we require information on actual exposure to and engagement with unreliable information. Moreover, while our study a different dataset and implements a different measure to identify user preferences for unreliable domains, we find results consistent with (*32*), engagement with unreliable domains is largely driven by user preferences, rather than search algorithms.

| Study | Method | Type | Focus | Keyword Focused | Num. Keywords | Search Engagement | Search Exposure |
|---|---|---|---|---|---|---|---|
| Greene et al | Real World | Academic | Unreliable Domains | No | - | Yes | Yes |
| (*32*) | Survey | Academic | Unreliable Domains | No | - | Yes | Yes |
| (*2*) | Survey | Academic | Unreliable Domains | No | - | Yes | No |
| (*3*) | Survey | Academic | Vaccine Misinformation | No | - | Yes | No |
| (*8*) | Audit | Media | Conspiracy | Yes | 8 | No | No |
| (*6*) | Audit | Academic | Misinformation | Yes | 13 | No | No |
| (*30*) | Audit | Academic | Conspiracy | Yes | 6 | No | No |
| (*29*) | Audit | Academic | Vaccine Misinformation | Yes | 4 | No | No |
| (*31*) | Audit | Academic | Election Misinformation | Yes | 20 | No | No |

Table S6: Comparison between the present study and prior research on search and unreliable media.

# Supplementary Text

## Results using Media Bias/Fact Check

In the following section, we reproduce the results from Figure 3 in the main paper, now replacing the quality rating from NewsGuard, with those compiled by Media Bias/Fact Check. These results are consistent with those presented in the main text. An outsized share of the exposure to unreliable sites originates from navigational searches, while the majority of the engagement does.

| | % Nav. Searches | % Exposure from Nav. | % Engagement from Nav. |
|---|---|---|---|
| Sample 1 | 1.62 | 44.40 | 84.67 |
| Sample 2 | 0.88 | 14.95 | 86.99 |

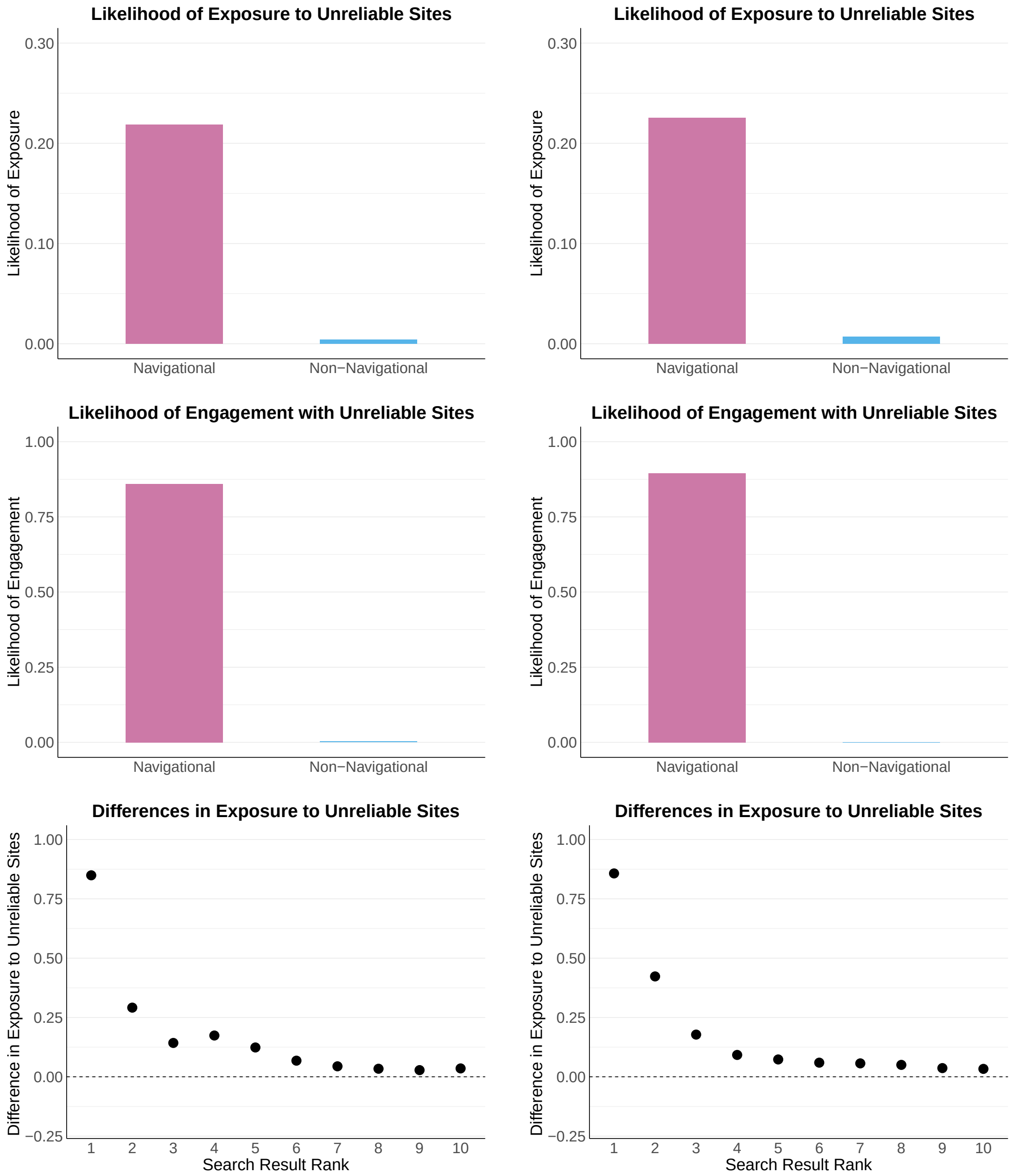


Figure S4: Exposure to and engagement with unreliable information sources for navigational and non-navigational searches. Engagement is the probability of engaging with unreliable sources conditional on the search query type. Exposure is the probability of being exposed to unreliable sources conditional on the search query type. **(A)** The proportion of total search results, exposure to unreliable sites, and engagement with unreliable sources originating from navigational searches. **(B&C)** The likelihood of exposure to unreliable sources across search types. **(D&E)** The likelihood of engagement with unreliable sources across search types. **(F&G)** Differences in exposure to unreliable sources at each result rank across search types.

## Exposure and Engagement Across Issue Areas

We identify search queries related to two important topics elections and health to explore potential heterogeneity in the impact of navigational searches. For elections, we identify a list of words related to elections (e.g. "voter", "elect") as well as phrases related to election denial (e.g. "rigged election"). Similarly for health, we identify a set of general health queries (e.g. "doctor", "medicare") as well as anti-vaccine terms. All dictionaries were created by consulting relevant media sources except the anti-vaccine keywords which were compiled by (*49*).

Table S7 replicates the table from the main paper for the set of queries relating to each topic. Overall these topics make up a very small percentage of the total search volume across both of our samples. While considerably less exposure or engagement with unreliable dominas originate from navigational searches, the amounts are fairly consistent across topics. The expectation being anti-vaccine terms.

Figure S5 replicates the figure from the main paper for the set of queries relating to each topic. In Sample 2 health related queries had the highest likelihood of being exposed to or engaging with unreliable domains. This was largely driven by searches about the supposed health benefits of various foods and supplements and false claims about politicians wanting to cancel Medicare.

| | Issue | % Nav. Searches | % Exposure from Nav. | % Engagement from Nav. |
|---|---|---|---|---|
| Sample 1 | Election | 0.04 | 0.20 | 0.08 |
| Sample 1 | Election Denial | 0.00 | 0.01 | 0.02 |
| Sample 1 | Health | 0.09 | 0.05 | 0.15 |
| Sample 1 | Anti-Vax | 0.86 | 3.10 | 7.56 |
| Sample 2 | Election | 0.02 | 0.01 | 0.07 |
| Sample 2 | Election Denial | 0.01 | 0.01 | 0.04 |
| Sample 2 | Health | 0.03 | 0.17 | 0.15 |
| Sample 2 | Anti-Vax | 0.60 | 1.55 | 1.25 |

Table S7: Exposure to and engagement with unreliable information sources for navigational searches across issue areas. The proportion of total search results, exposure to unreliable sites, and engagement with unreliable sources originating from navigational searches.

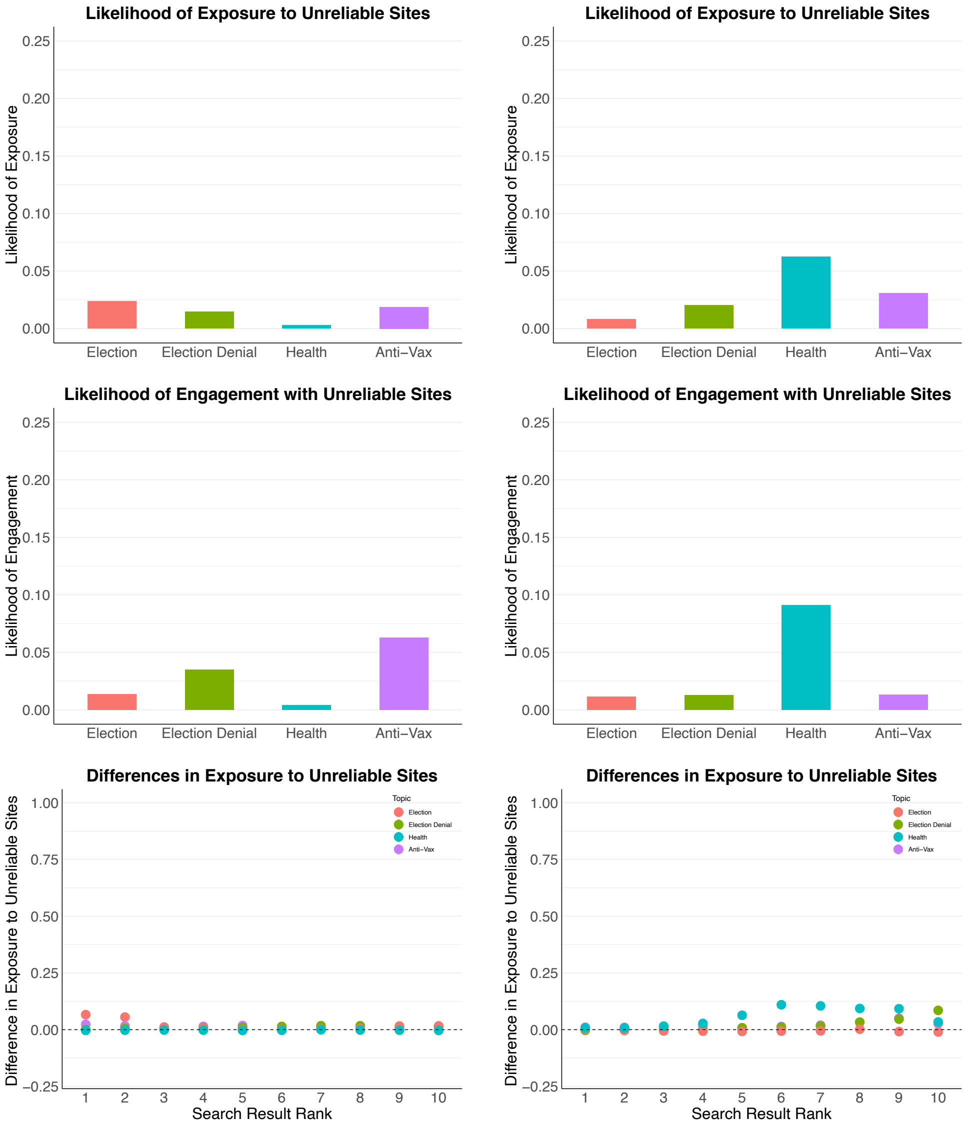


Figure S5: Exposure to and engagement with unreliable information sources across issue areas. Engagement is the probability of engaging with unreliable sources conditional on the search query type. Exposure is the probability of being exposed to unreliable sources conditional on the search query type. **(A&B)** The likelihood of exposure to unreliable sources across search types. **(C&D)** The likelihood of engagement with unreliable sources across search types. **(E&F)** Differences in exposure to unreliable sources at each result rank across search types.

## REFERENCES AND NOTES


1. K. Purcell, L. Rainie, J. Brenner, Search engine use 2012, *Pew Internet & American Life Project* (2012); https://pewresearch.org/internet/2012/03/09/search-engine-use-2012/.

2. A. M. Guess, B. Nyhan, J. Reifler, Exposure to untrustworthy websites in the 2016 us election. *Nat. Hum. Behav.* **4**, 472–480 (2020).

3. A. M. Guess, B. Nyhan, Z. O'Keeffe, J. Reifler, The sources and correlates of exposure to vaccine-related (mis) information online. *Vaccine* **38**, 7799–7805 (2020).

4. M. Chalkiadakis, A. Kornilakis, P. Papadopoulos, E. Markatos, N. Kourtellis, The rise and fall of fake news sites: A traffic analysis, *13th ACM Web Science Conference 2021* (ACM, 2021), pp. 168–177.

5. D. Wakabayashi, M. Uong, D. North, As Google fights fake news, voices on the margins raise alarm, *The New York Times* (2017); https://nytimes.com/2017/09/26/technology/google-search-bias-claims.html.

6. D. Bush, A. Zaheer, Bing's top search results contain an alarming amount of disinformation, *Internet Observatory News* (2019); https://cyber.fsi.stanford.edu/io/news/bing-search-disinformation.

7. F. Tripodi, Google search is quietly damaging democracy, *Wired* (2022); https://wired.com/story/google-search-quietly-damaging-democracy/.

8. S. Thompson, Fed up with Google, conspiracy theorists turn to DuckDuckGo, *The New York Times* (2022); https://nytimes.com/2022/02/23/technology/duckduckgo-conspiracy-theories.html.

9. K. Clayton, N. T. Davis, B. Nyhan, E. Porter, T. J. Ryan, T. J. Wood, Elite rhetoric can undermine democratic norms. *Proc. Natl. Acad. Sci. U.S.A.* **118**, e2024125118 (2021).

10. N. Berlinski, M. Doyle, A. M. Guess, G. Levy, B. Lyons, J. M. Montgomery, B. Nyhan, J. Reifler, The effects of unsubstantiated claims of voter fraud on confidence in elections. *J. Exp. Political Sci.* **10**, 1–16 (2021).

11. K. Clayton, C. Finley, D. Flynn, M. Graves, B. Nyhan, Evaluating the effects of vaccine messaging on immunization intentions and behavior: Evidence from two randomized controlled trials in vermont. *Vaccine* **39**, 5909–5917 (2021).

12. D. M. West, The role of misinformation in Trump's insurrection, *Brookings Institution* (2021); https://brookings.edu/articles/the-role-of-misinformation-in-trumps-insurrection/.

13. S. Mcammon, L. Baker, Disinformation fuels distrust and even violence at all levels of government, *NPR* (2021); https://npr.org/2021/03/01/971436680/from-the-u-s-capitol-to-local-governments-disinformation-disrupts.

14. H. Hosseinmardi, A. Ghasemian, A. Clauset, M. Mobius, D. M. Rothschild, D. J. Watts, Examining the consumption of radical content on YouTube. *Proc. Natl. Acad. Sci. U.S.A.* **118**, e2101967118 (2021).

15. K. Munger, J. Phillips, Right-wing Youtube: A supply and demand perspective. *Int. J. Press Politics* **27**, 186–219 (2022).

16. A. Y. Chen, B. Nyhan, J. Reifler, R. E. Robertson, C. Wilson, Subscriptions and external links help drive resentful users to alternative and extremist youtube channels. *Sci. Adv.* **9**, eadd8080 (2023).

17. S. Vosoughi, D. Roy, S. Aral, The spread of true and false news online. *Science* **359**, 1146–1151 (2018).

18. W. H. Dutton, B. Reisdorf, E. Dubois, G. Blank, Search and politics: The uses and impacts of search in Britain, France, Germany, Italy, Poland, Spain, and the United States, *Quello Center Working Paper* (2017); https://papers.ssrn.com/sol3/papers.cfm?abstract_id=2960697.

19. D. Trielli, N. Diakopoulos, Search as news curator: The role of google in shaping attention to news information, *Proceedings of the 2019 CHI Conference on Human Factors in Computing Systems* (2019), pp. 1–15.

20. B. Edelman, 2022 edelman trust barometer, *Edelman Trust Barometer* (2022); https://edelman.com/trust/2022-trust-barometer.

21. R. Epstein, R. E. Robertson, The search engine manipulation effect (SEME) and its possible impact on the outcomes of elections. *Proc. Natl. Acad. Sci. U.S.A.* **112**, E4512-21 (2015).

22. R. Epstein, R. E. Robertson, D. Lazer, C. Wilson, Suppressing the search engine manipulation effect (seme). *Proc. ACM Hum. Comput. Interact.* **1**, 1–22 (2017).

23. R. E. Robertson, S. Jiang, K. Joseph, L. Friedland, D. Lazer, C. Wilson, Auditing partisan audience bias within google search. *Proc. ACM Hum. Comput. Interact.* **2**, 1–22 (2018).

24. J. Kulshrestha, M. Eslami, J. Messias, M. B. Zafar, S. Ghosh, K. P. Gummadi, K. Karahalios, Search bias quantification: Investigating political bias in social media and web search. *Inf. Retr. J* **22**, 188–227 (2019).

25. D. Hu, S. Jiang, R. E. Robertson, C. Wilson, "Auditing the partisanship of google search snippets," in *the Proceedings of the World Wide Web Conference* (WWW, 2019), pp. 693–704.

26. C. Puschmann, Beyond the bubble: Assessing the diversity of political search results. *Digit. Journal.* **7**, 824–843 (2019).

27. A. Urman, M. Makhortykh, R. Ulloa, The matter of chance: Auditing web search results related to the 2020 US presidential primary elections across six search engines. *Soc. Sci. Comput. Rev.* **40**, 1323–1339 (2022).

28. P. T. Metaxas, Y. Pruksachatkun, "Manipulation of search engine results during the 2016 us congressional elections," in the *Proceedings of the ICIW* (ICW, 2017).

29. P. Ghezzi, P. G. Bannister, G. Casino, A. Catalani, M. Goldman, J. Morley, M. Neunez, A. Prados-Bo, P. R. Smeesters, M. Taddeo, Online information of vaccines: Information quality, not only privacy, is an ethical responsibility of search engines. *Front. Med.* **7**, 400 (2020).

30. A. Urman, M. Makhortykh, R. Ulloa, J. Kulshrestha, Where the earth is flat and 9/11 is an inside job: A comparative algorithm audit of conspiratorial information in web search results. *Telemat. Inform.* **72**, 101860 (2022).

31. H. Zade, M. Wack, Y. Zhang, K. Starbird, R. Calo, J. Young, J. D. West, Auditing Google's search headlines as a potential gateway to misleading content: Evidence from the 2020 US election. *J. Online Trust. Saf.* **1**, 4 (2022).

32. R. E. Robertson, J. Green, D. J. Ruck, K. Ognyanova, C. Wilson, D. Lazer, Users choose to engage with more partisan news than they are exposed to on google search. *Nature* **618**, 342–348 (2023).

33. E. Bakshy, S. Messing, L. A. Adamic, Exposure to ideologically diverse news and opinion on Facebook. *Science* **348**, 1130–1132 (2015).

34. K. Garimella, T. Smith, R. Weiss, R. West, Political polarization in online news consumption. *Proc. Int. AAAI Conf. Web. Soc. Media* **15**, 152–162 (2021).

35. NewsGuard, Fighting misinformation with journalism, not algorithms (2021).

36. K. Aslett, A. M. Guess, R. Bonneau, J. Nagler, J. A. Tucker, News credibility labels have limited average effects on news diet quality and fail to reduce misperceptions. *Sci. Adv.* **8**, eabl3844 (2022).

37. B. Pan, H. Hembrooke, T. Joachims, L. Lorigo, G. Gay, L. Granka, In Google we trust: Users' decisions on rank, position, and relevance, *J. Comput. Mediat. Commun.* **12**, 801–823 (2007).

38. A. Broder, A taxonomy of web search. *ACM SIGIR Forum* **36**, 3–10 (2002).

39. B. J. Jansen, D. L. Booth, A. Spink, Determining the informational, navigational, and transactional intent of web queries. *Inf. Process. Manag.* **44**, 1251–1266 (2008).

40. D. Trielli, N. Diakopoulos, Partisan search behavior and Google results in the 2018 US midterm elections. *Inform. Commun. Soc.* **25**, 145–161 (2022).

41. A. A. Arechar, J. Allen, A. J. Berinsky, R. Cole, Z. Epstein, K. Garimella, A. Gully, J. G. Lu, R. M. Ross, M. N. Stagnaro, Y. Zhang, G. Pennycook, D. G. Rand, Understanding and combatting misinformation across 16 countries on six continents. *Nat. Hum. Behav.* **7**, 1502–1513 (2023).

42. B. Nyhan, Why the backfire effect does not explain the durability of political misperceptions, *Proc. Natl. Acad. Sci. U.S.A.* **118**, e1912440117 (2021).

43. J. M. Carey, A. M. Guess, P. J. Loewen, E. Merkley, B. Nyhan, J. B. Phillips, J. Reifler, The ephemeral effects of fact-checks on covid-19 misperceptions in the United States, Great Britain and Canada. *Nat. Hum. Behav.* **6**, 236–243 (2022).

44. N. Grinberg, K. Joseph, L. Friedland, B. Swire-Thompson, D. Lazer, Fake news on Twitter during the 2016 US presidential election. *Science* **363**, 374–378 (2019).

45. H. Lin, J. Lasser, S. Lewandowsky, R. Cole, A. Gully, D. G. Rand, G. Pennycook, High level of correspondence across different news domain quality rating sets. *PNAS Nexus* **2**, pgad286 (2023).

46. A. Hannak, P. Sapiezynski, A. Molavi Kakhki, B. Krishnamurthy, D. Lazer, A. Mislove, C. Wilson, "Measuring personalization of web search," in *Proceedings of the 22nd International Conference on World Wide Web* (ACM, 2013), pp. 527–538.

47. C. Petersen, J. G. Simonsen, C. Lioma, Power law distributions in information retrieval. *ACM Trans. Inf. Syst.* **34**, 1–37 (2016).

48. D. M. Lazer, M. A. Baum, Y. Benkler, A. J. Berinsky, K. M. Greenhill, F. Menczer, M. J. Metzger, B. Nyhan, G. Pennycook, D. Rothschild, The science of fake news. *Science* **359**, 1094–1096 (2018).

49. T. Mitts, N. Pisharody, J. Shapiro, "Removal of anti-vaccine content impacts social media discourse," in *Proceedings of the 14th ACM Web Science Conference 2022* (ACM, 2022), pp. 319–326.
